# Early fluid resuscitation with hydroxyethyl starch 130/0.4 (6%) in severe burn injury: a randomized, controlled, double-blind clinical trial

**DOI:** 10.1186/cc13168

**Published:** 2013-12-23

**Authors:** Markus Béchir, Milo A Puhan, Mario Fasshauer, Reto A Schuepbach, Reto Stocker, Thomas A Neff

**Affiliations:** 1 Surgical Intensive Care Medicine, University Hospital of Zurich, Raemistrasse 100, Zurich CH-8091, Switzerland; 2 Swiss Paraplegic Center, Department of Intensive Care, Pain and Operative Medicine, Guido A. Zäch Strasse 1, Nottwil 6207, Switzerland; 3 Institute for Social and Preventive Medicine, University of Zurich, Zurich 8001, Switzerland; 4 Department of Anesthesia & Intensive Care Medicine, Cantonal Hospital of Muensterlingen, Spitalcampus 1, Muensterlingen 8596, Switzerland

## Abstract

**Introduction:**

There are limited data on the efficacy of early fluid resuscitation with third-generation hydroxyethyl starch (HES 130) in burn injury. Adverse effects of HES on survival and organ function have been reported.

**Methods:**

In this randomized, controlled, double-blind trial, 48 patients with severe burn injury were assigned to receive either lactated Ringer’s solution plus 6% HES 130/0.4 in a ratio of 2:1 or lactated Ringer’s solution with no colloid supplement for the first 72 hours. Primary outcome parameter was the group difference of administered total fluid from intensive care unit (ICU) admission up to day 3. Secondary outcomes included kidney and lung injury and failure, length of stay, and mortality.

**Results:**

Three-day totals of administered resuscitation fluid (medians) were 21,190 mL in the lactated Ringer’s group and 19,535 mL in the HES group (HES: −1,213 mL; *P* = 0.39). Creatinine levels from day 1 to 3 (HES: +0.4 μmol/L; 95% confidence interval (CI) −18.7 to 19.5; *P* = 0.97) and urinary outputs from day 1 to 3 (HES: −58 mL; 95% CI −400 to 283; *P* = 0.90) were not different. Six patients in each group developed acute respiratory distress syndrome (ARDS) (risk ratio 0.96; 95% CI 0.35 to 2.64; *P* = 0.95). Length of ICU stay (HES vs. lactated Ringer’s: 28 vs. 24 days; *P* = 0.80) and length of hospital stay (31 vs. 29 days; *P* = 0.57) were similar. Twenty-eight-day mortality was 4 patients in each group (risk ratio 0.96; 95% CI 0.27 to 4.45; *P* = 0.95), and in-hospital mortality was 8 in the HES group vs. 5 patients in the lactated Ringer’s group (hazard ratio 1.86; 95% CI 0.56 to 6.19; *P* = 0.31).

**Conclusions:**

There was no evidence that early fluid resuscitation with balanced HES 130/0.4 (6%) in addition to lactated Ringer’s solution would lead to a volume-sparing effect in severe burn injury. Together with the findings that early renal function, incidence of ARDS, length of stay, and mortality were not negatively influenced by HES in this setting, balanced HES 130/0.4 (6%) plus lactated Ringer’s solution could not be considered superior to lactated Ringer’s solution alone.

**Trial registration:**

ClinicalTrials.gov NCT01012648

## Introduction

There is an ongoing debate on fluid resuscitation in severe burn injury, especially for the first 24 hours after trauma. Aggressive intravenous fluid therapy according to the Baxter formula is a mainstay of initial therapy. Multiple pathophysiological changes characterize the early post-traumatic phase. Massive systemic inflammation comparable to severe sepsis leads by the release of numerous mediators such as leukotrienes, prostaglandins, and particularly histamine, in combination with complement activation products, to an extensive capillary leak [1,2]. Intravascular molecule and fluid shifts into the extravascular space cause severe hypovolemia and shock [3]. Changes in capillary membrane permeability also produce electrolytic alteration with intracellular sodium accumulation and consecutive cellular swelling [4]. Excessive tissue edema, promoted to a large extent by the leakage of plasma proteins into the extravascular space, normally occurs within the first few hours after trauma. The capillary leak is believed to resolve within 8 to 24 hours after trauma, but data vary [1,5]. In this critical situation of massive inflammation, hypovolemia, and large edema formation, it remains unclear whether a “crystalloid only” therapy or a combination of crystalloids plus colloids should be used for volume resuscitation. Expert opinion consisted of strictly avoiding colloids such as hydroxyethyl starches (HESs) during the first 24 hours [6]. This restriction was based on reports from the early 1970s expressing the fear of overloading the interstitial compartment with colloids in the early stage of trauma due to increased capillary leakage, thus leading to impaired wound healing after surgical treatment [7,8]. Although in 1998 the Cochrane Injuries Group presented a relative risk of death after albumin administration of 2.4 in a meta-analysis [9], human albumin is still used in burn to reduce the fluid requirements for resuscitation [10,11] and tissue edema. With the same intention, different types of HES are frequently administered in burn injury, although safety and efficacy of HES products for fluid resuscitation are not fully evaluated and are intensely disputed especially during the last few years. However, we just recently have demonstrated in a prospective interventional open-label study that hyperoncotic HES 200/0.5 (10%) might be associated with fatal outcome when used for early fluid resuscitation in severe burn injury [12]. A recent randomized controlled trial assigned 26 burn patients either to Hartmann’s solution plus HES 200/0.6 (6%) or to Hartmann’s solution only. The HES-supplemented fluid therapy led to significantly less fluid application than the Hartmann’s regimen and showed reduced interstitial edema [13]. The least side effects on kidney function and coagulation are attributed to the third-generation HESs such as HES 130/0.4 (6%), but data on early fluid resuscitation in major burn with these modern starches are limited. Therefore in this current randomized controlled trial we addressed the question of whether modern HES 130/0.4 (6%) administered within the first 24 hours after severe burn injury and up to 72 hours of treatment would be able to show any fluid-sparing effect.

(ClinicalTrials.gov number, NCT01012648).

## Materials and methods

### Trial design

This study was an investigator-initiated, prospective, randomized, controlled, double-blind single-center trial. The study protocol was approved by the local ethical committee (KEK Kantonale Ethik Kommission [Cantonal Ethical Committee] 4, Canton Zurich) and the Swiss Agency for Therapeutic Products (Swissmedic). Power calculation and planning of the statistical analysis were done at the Horten Centre for patient-oriented research at University Hospital of Zurich. Reporting of the study was done according to the CONSORT (Consolidated Standards of Reporting Trials) guidelines.

### Participants

All adults (age ≥16 years) who had 2nd- or 3rd-degree acute burn injury and more than 15% of body surface area burned and who were admitted to the University Hospital of Zurich burn unit between 1 November 2009 and 31 January 2013 were eligible for the study. All necessary written informed consent (deferred consent, if necessary, according to Swiss law HMG § 55 and § 56) was obtained from the patient or their legal surrogate within 24 hours after inclusion. In the case of written consent of the legal surrogate, all survivors gave written informed consent after recovery, which is in line with the local ethics committee regulations. Patients were excluded when they were expected to succumb within the next 24 to 36 hours (that is, burn victims with whole body burn trauma) or in situations of palliative care, pregnancy, lack of informed consent, known allergy to HES, contraindications for balanced 6% HES 130/0.4, intracerebral bleeding, acute renal failure, severe hypernatremia and other severe electrolyte disorders, severe von Willebrand Syndrome, and acute liver failure.

### Study setting

The study was performed in a tertiary burn unit at the University Hospital of Zurich, Switzerland. The center is the larger of the two national burn units in Switzerland and runs six acute care beds. About 80 severe burn victims are admitted to the university hospital per year.

### Interventions

**Figure 1:**
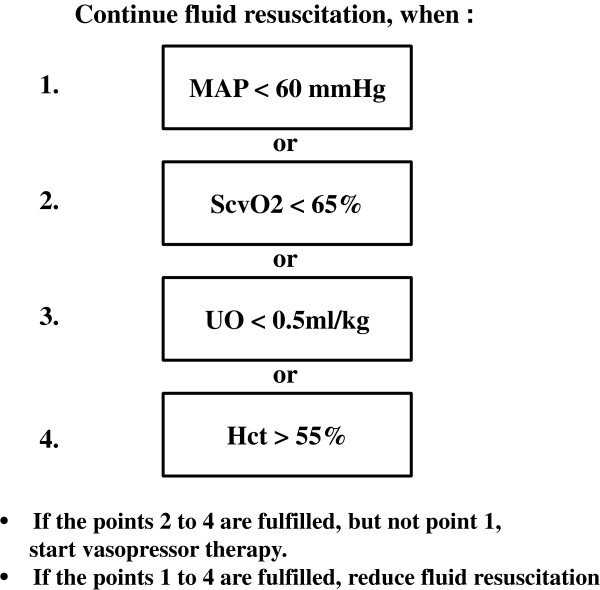
**Hct, hematocrit; MAP, mean arterial pressure; ScvO**_**2**_**, central venous oxygen saturation; UO, urinary output.**

The primary study medication was balanced 6% HES 130/0.4. After patient enrollment and randomization, fluid resuscitation was done as follows. Each patient first received two bags of unblinded lactated Ringer’s solution (500 mL each bag), followed by one bag (500 mL) of blinded study solution, the latter being either again lactated Ringer’s solution or balanced 6% HES 130/0.4. After each bag of study solution, all patients again received two bags of unblinded lactated Ringer’s solution, before a next bag of study solution from the blinded box was infused. This fluid regimen alternating unblinded lactated Ringer’s fluid with blinded study solution ensured an overall ratio of crystalloids versus colloids of 2:1 in the HES patients. The patients not receiving HES but blinded lactated Ringer’s study solution instead were exposed solely to crystalloids during the entire course of the study. Fluid resuscitation was guided by predefined target variables as listed in Figure 1. Accordingly, fluid administration was increased or decreased until target variables were reached. The administration of additional albumin or any other colloid was excluded in both groups. All resuscitation fluids were administered as continuous infusions via peristaltic pumps. Infusion rate (mL/h) was continuously adjusted to the actual patient fluid needs. Except for volume resuscitation, there was no difference in patient care, including cardiovascular monitoring, pharmacologic and respiratory support, nutrition, and surgical treatment of burn wounds.

### Outcome measures

Primary outcome was the group difference of administered resuscitation fluid within the first 72 hours after admission. Secondary endpoints were creatinine level at day 1 and the difference over day 1 to 3, urine output at day 1 and the difference over day 1 to 3, incidence of acute respiratory distress syndrome (ARDS) [14] during hospitalization, length of stay in the intensive care unit (ICU), length of stay in the hospital, in-hospital mortality, and 28-day mortality. Collected baseline characteristics were age, sex, systolic and diastolic blood pressure, heart rate, body weight, body height, percentage of burn, and the amount of pre-hospital administered fluids.

A *post hoc* analysis was performed for 90-day mortality and incidence of renal replacement therapy during hospital stay.

### Sample size

We based our sample size calculations on the primary outcome, which is the total volume of fluids given within the first 72 hours of treatment. We used data from our previous study with HES 200/0.5 [12] to estimate the average total volume of fluids given within the first 72 hours of treatment with crystalloids (25 L with crystalloid and 18 L with HES) and to estimate its variability (standard deviation of around 12 and 7 L, respectively). A sample size of 24 patients in each group allowed a difference of 25%, respectively, in total volume of fluids given within the first 72 hours of treatment between the groups with a power of 80% at a significance level of 5% (two-sided).

### Blinding and randomization

A third party not involved in the conduction (KAZ, Kantonsapotheke, Zurich) performed randomization and prepared the study solution, either balanced 6% HES 130/0.4 or lactated Ringer’s solution, by sealing the identical 500-mL bags in black plastic foil concealing the product label and content. Thus, there was no possibility to recognize the fluid used. Bags were packed into boxes. Three boxes of the same content labelled in consecutive order were assigned to each patient, one box for each 24-hour period up to 72 hours. For randomization, minimization was used with stratification for age (< or ≥50 years). Since minimization does not have a pre-specified randomization list, concealment of random allocation was ensured. Thus, all patients were randomly assigned double-blind either to the lactated Ringer’s plus balanced 6% HES 130/0.4 group or to the lactated Ringer’s-only group, and study medication was assigned to the patients. To make sure that there was no overload of 6% HES 130/0.4 study medication, the maximum amount (for HES according to the manufacturer’s manual 50 mL per kg body weight per 24 hours) was calculated on the basis of the estimated body weight at study enrollment. A body weight of 80 kg, for example, led to 4,000 mL of study solution, which resulted in eight bags of 500 mL each of blinded solution. Not more than that was brought to the patient.

### Statistical methods

We included all patients in the analysis according to the group they were randomly assigned to. To compare the outcomes between the two groups, we used linear regression analysis for continuous outcomes (for example, fluids), logistic regression for binary outcomes (for example, ARDS), and Cox proportional hazard regression for in-hospital mortality, always with group allocation as an independent variable. To compare the total volume of fluids given within the first three days after randomization, creatinine values, and urinary output over the first 72 hours, we used a random effects model that took the auto-correlated structure of repeated measurements (measurement on first, second, and third day) into consideration (xtreg command of STATA). All analyses were conducted by using STATA (STATA for Windows, version 10.2; Stata Corp., College Station, TX, USA).

## Results

### Participants

**Figure 2:**
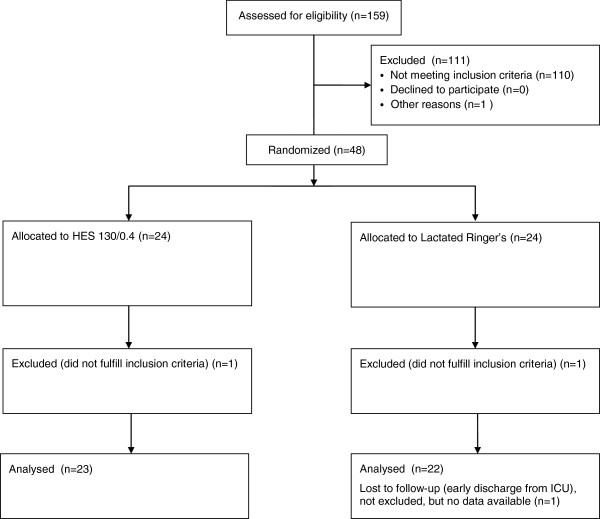
Study flow diagram.

From 1 November 2009 through 31 January 2013, 159 patients were assessed for inclusion in the study, and 111 patients were not eligible. From the enrolled 48 patients, 24 patients were assigned to receive lactated Ringer’s solution plus balanced 6% HES 130/0.4 (HES group) and 24 to receive lactated Ringer’s solution (lactated Ringer’s group). Two patients had to be excluded from the study because they retrospectively did not fulfill inclusion criteria. (One primarily included patient with a negative pregnancy test had to be excluded secondarily because a revised pregnancy test was positive shortly thereafter. One patient was initially assessed as having a 20% deep burned area but showed less than 15% intraoperatively). One patient was lost for analysis in the lactated Ringer’s group because of early discharge within less than 72 hours from the ICU. This patient was formally not excluded, but no study data were available for incorporation in the statistics. Participant flow is shown in Figure 2. Baseline characteristics were well balanced between groups (Table 1).

**Table 1:** Baseline characteristics of the patients

**Characteristic**	**HES (n = 23)**	**Lactated Ringer’s (n = 22)**
Age, years	49 (22, 69)	47 (26, 61)
Sex, male	17 (73.9%)	17 (77.2%)
Systolic blood pressure, mm Hg	109 (93, 130)	123 (104, 150)
Diastolic blood pressure, mm Hg	60 (55, 65)	68 (59, 76)
Heart rate, beats/min	83 (70, 95)	86 (75, 95)
Weight, kg	75 (70, 83)	80 (70, 80)
Height, cm	175 (170, 180)	176 (170, 180)
Burned TBSA, %	31 (21, 47)	32 (20, 50)

Data are represented as median (25th and 75th percentiles) or median (percentage). None of the differences between the two groups was significant (*P* >0.05). Two patients were randomly assigned but were excluded because they did not fulfill inclusion criteria, and one patient was not excluded formally but was not in analyses, because of a lack of data. HES, hydroxyethyl starch; TBSA, total body surface area.

### Fluid therapy

During the pre-hospital phase, the lactated Ringer’s group received a median of 1,800 mL and the HES group 2,000 mL of fluid (difference not significant); no colloids were administered. Calculated fluid requirement for the first 24 hours based on the Baxter formula was not different between the two groups (lactated Ringer’s group: 8,520 versus HES: 9,000 mL). The median 3-day total of effectively administered fluid was 21,190 mL in the lactated Ringer’s group versus 19,535 mL in the HES group. A median amount of 5,650 mL of HES was administered in the colloid group, and no HES was administered in the lactated Ringer’s group (Table 2).

**Table 2:** Fluid therapy

**Variable**	**HES (n = 23)**	**Lactated Ringer’s (n = 22)**
Pre-hospital crystalloids, mL	2,000 (1,000, 2,500)	1,800 (1,000, 3,600)
Pre-hospital colloids, mL	0 (0, 0)	0 (0, 0)
Baxter formula, mL	9,000 (5,880, 13,536)	8,520 (7,920, 18,080)
Total fluids at day 1, mL	10,050 (6,700, 16,800)	11,575 (9,300, 19,770)
Total fluids at day 2, mL	5,500 (3,750, 8,825)	5,025 (3,180, 9,300)
Total fluids at day 3, mL	3,340 (2,060, 7,000)	4,150 (1,640, 6,100)
Total fluids at days 1–3, mL	19,535 (13,820, 29,770)	21,190 (14,760, 33,960)
Total crystalloids at days 1–3, mL	13,200 (10,075, 19,020)	21,190 (14,760, 33,960)
Total colloids at days 1–3, mL	5,650 (3,745, 9,000)	0 (0, 0)

Data are represented as median (25th and 75th percentiles). HES, hydroxyethyl starch.

### Outcomes

Regarding the primary endpoint, there was a group difference in fluids given over the first 72 hours of −1,213 mL in the HES group, which was not statistically significant (95% confidence interval (CI) −3,975 to 1,549; *P* = 0.39). With regard to secondary outcomes, there was no difference over the first 72 hours in creatinine levels (+0.4 μmol/L; 95% CI −18.7 to 19.5; *P* = 0.97) or in urinary output (−58 mL; 95% CI −400 to 283; p = 0.90). The incidence of ARDS was 6 patients in each group (risk ratio 0.96; 95% CI 0.35 to 2.64; *P* = 0.95), and again there was no difference in length of ICU stay and hospital stay (28 vs. 24 days; *P* = 0.80 and 31 vs. 29 days; *P* = 0.57), respectively. Twenty-eight-day mortality was 4 patients in each group (risk ratio 0.96; 95% CI 0.27 to 4.45; *P* = 0.95), and in-hospital mortality was 8 in the HES group versus 5 in the lactated Ringer’s group (hazard ratio 1.86; 95% CI 0.56 to 6.19; *P* = 0.31) (Table 3).

**Table 3:** Primary and secondary outcomes

**Outcome**	**HES (n = 23)**	**Lactated Ringer’s (n = 22)**	**Difference**	***P *** **value**
Primary outcome				
Total volume at days 1–3, mL			−1,213 (95% CI −3,975 to 1,549)	0.39
Secondary outcomes				
Creatinine at day 1, μmol/L	77 (66, 99)	74 (55, 90)		
Creatinine at days 1–3, μmol/L			0.4 (95% CI −18.7 to 19.5)	0.97
Urinary output at day 1, mL/d	1,360 (1,020, 1,770)	1,430 (970, 2,225)		
Urinary output at days 1–3, mL			−58 (95% CI −400 to 283)	0.90
Incidence of ARDS	6 (26.1%)	6 (27.3%)		
Risk ratio for ARDS with HES			0.96 (95% CI 0.35 to 2.64)	0.95
28-day mortality	4 (17.4%)	4 (18.2%)		
Risk ratio for 28-day mortality with HES			0.96 (95% CI 0.27 to 4.45)	0.95
In hospital mortality	8 (34.8%)	5 (22.7%)		
Hazard ratio for in-hospital death with HES			1.86 (95% CI 0.56 to 6.19)	0.31
Length of stay in ICU, days	28 (10, 58)	24 (11, 49)		0.80
Length of stay in hospital, days	31 (18, 58)	29 (14, 61)		0.57

Data are represented as median (25th and 75th percentiles) or number of patients (percentage) or risk ratio (confidence interval) or hazard ratio (confidence intervals, or CIs). For total volume, creatinine, and urinary output over days 1–3, absolute values with original units (confidence intervals) are depicted. ARDS, acute respiratory distress syndrome; HES, hydroxyethyl starch; ICU, intensive care unit.

The results of the *post hoc* analysis of 90-day mortality and incidence of renal replacement therapy showed no difference between the groups. Data are depicted in Table 4.

**Table 4:** ***Post hoc *** **analysis 90-day mortality and need for renal replacement therapy**

**Outcome (*post hoc *** **analysis)**	**HES (n = 23)**	**Lactated Ringer’s (n = 22)**	**Difference**	***P *** **value**
90-day mortality	8 (34.8%)	6 (27.3%)		
Risk ratio for 90-day mortality with HES			1.27 (95% CI 0.51 to 3.26)	0.59
Need for RRT	6 (26.1%)	6 (27.3%)		
Risk ratio for need of RRT with HES			0.96 (95% CI 0.35 to 2.64)	0.95

Data are represented as number of patients (percentage) or risk ratio (confidence interval, or CI). HES, hydroxyethyl starch; RRT, renal replacement therapy.

## Discussion

In this randomized controlled trial, no fluid saving effect was detected by the use of balanced 6% HES 130/0.4 as compared with lactated Ringer’s solution alone in patients with severe burn injury. Furthermore, early renal function as determined by serum creatinine levels, development of ARDS, length of ICU and hospital stay, and in-hospital and 28-day mortality were not different between treatment groups.

In severe burn injury with massive systemic inflammation comparable to severe sepsis, aggressive fluid resuscitation to maintain hemodynamic stability and stable kidney function is pivotal. The most widely accepted formula to estimate fluid requirements in burns is the Baxter formula, which, however, rather underestimates the volume needed in about half of the patients [15-17]. The downside of significant fluid load in burned patients might be accentuated edema formation and thus impaired wound healing after surgical treatment. Hence, a reduction of fluid load, especially during the first 24 to 48 hours, when the most resuscitation volume is needed, appears to be desirable in order to improve surgical outcome.

The role of HES in various clinical settings remains controversial. A possible volume-sparing effect, assigned to colloids in general, is the main indication for its widespread use, although the extent of fluid load reduction may be overestimated. There are only few data about the use of HES in patients with burn injury. In a recent randomized controlled trial in burned patients, Vlachou and colleagues [13] showed a clear volume-sparing effect and furthermore reduced edema formation with HES 200/0.6 (6%) supplementation. However, as reported by our group, older-generation HES such as the hyperoncotic HES 200/0.6 (10%) might be associated with a higher incidence of renal failure and higher overall mortality in severe burn injury [12]. One explanation could be related to the fact that only about 33% to 66% of the administered hyperoncotic HES is excreted in the urine in the first 24 hours after infusion [18]. Thus, the remaining HES molecules, which are still in high concentration, may circulate for a long time and a substantial proportion might accumulate in various tissues, including kidney. Hyperoncotic HES deposition was demonstrated in dogs by histopathology in intravascular and interstitial spaces of various organs, including proximal renal tubular cells, thus possibly inducing renal failure [19]. There are also many case studies describing acute deterioration of pre-existing renal impairment after the administration of hyperoncotic HES [20,21].

Very limited data are available on modern third-generation HESs such as HES 130 in burns. Only one small randomized open-label study reported more favorable parameters related to the extent of tissue edema and a reduced mortality with HES 130/0.4 [13]. With regard to kidney function, James and colleagues [22] just recently demonstrated in penetrating trauma patients resuscitated with HES 130/0.4 a better lactate clearance and less acute kidney injury than in patients treated with saline. Furthermore, in an observational retrospective study in 363 ICU patients, Boussekey and colleagues [23] showed no difference in acute kidney injury after the use of HES 130/0.4 as compared with crystalloids. These findings, not necessarily connecting the administration of HES 130/0.4 to acute renal failure, support our current data showing no increasing creatinine levels over the first 3 days of fluid resuscitation with HES 130. However, it has to be mentioned that in our study HES 130 was co-infused together with lactated Ringer’s solution in a ratio of 2:1, which might protect the kidney from acute deterioration and failure. In large contrast, several recent trials and analyses not focusing on burn injury have drawn different conclusions with regard to kidney failure. Although an improvement of sublingual microcirculation after resuscitation with HES 130/0.4 versus saline was reported [24], septic patients receiving HES 130 were more likely to develop acute kidney injury, requiring renal replacement therapy, and to be at increased risk of death after 90 days [25]. This is in line with the findings in a large multicenter trial in which it was shown in 7,000 patients that the application of HES 130/0.4 resulted in more adverse events and more renal replacement therapies as compared with patients receiving 0.9% saline for fluid resuscitation [26]. When these recent large studies and meta-analyses are taken together, HES products, including HES 130 preparations, might be associated with increased mortality and acute kidney injury in ICU patients [27-32]. Whether these data can ultimately be translated to burn injury needs further investigation.

The current study has several limitations: firstly, the application of resuscitation fluids was algorithm-based and conducted by different members of our ICU staff. Nevertheless, over two years of study duration, this effect has probably been levelled out over time. Secondly, the used volume resuscitation algorithm was very traditional and did not include any hemodynamic measurement tools such as pulmonary artery catheter or pulse-induced contour cardiac output. Only clinical signs and various hemodynamic and surrogate parameters (mean arterial pressure, central venous oxygen saturation, urinary output, and hematocrit) were used to guide volume therapy. The reason for this simplified approach comes on the one hand from our clinical experience with fluid therapy in burned patients suggesting that this approach is reliable and on the other hand from the lack of clear evidence for better fluid therapy by the use of advanced hemodynamic guidance tools. Our data show that estimated fluid requirements for the first 24 hours (calculated with the Baxter formula) are comparable to the effectively infused amount of resuscitation fluid in both the HES and the crystalloid group. And as known from the literature, the Baxter formula rather underestimates the necessary amount of infusion fluid [15-17], which was the case in our study as well. Thirdly, power calculation was done to detect a potential volume-sparing effect but not to determine differences in mortality, organ failure, and length of stay. The latter are secondary endpoints, for which the study is underpowered due to the relatively small sample size. Therefore, differences in mortality, organ failure, and length of stay have to be interpreted with caution.

The strength of the study is its randomized, double-blinded, and controlled design, making the findings reliable and of clinical relevance. An implication for further research would be the initiation of randomized controlled trials with large sample sizes to strengthen the current evidence of a missing volume-sparing effect of modern HESs in burn injury. With regard to safety concerns that have arisen after the latest meta-analysis reporting a significant risk for mortality and acute kidney injury in various patient groups [28], further studies should specifically address this issue in burned patients.

## Conclusions

This randomized, controlled, double-blind study did not provide any evidence that early fluid resuscitation with balanced HES 130/0.4 (6%) as an add-on fluid to lactated Ringer’s solution during the first 72 hours after burn injury would lead to a volume-sparing effect. Together with the findings that early renal function, incidence of ARDS, length of stay, and mortality were not negatively influenced by HES in this setting, balanced HES 130/0.4 (6%) plus lactated Ringer’s solution could not be considered superior to lactated Ringer’s solution alone.

## Key messages

● All patients received more resuscitation fluid than Baxter formula suggested.

● The use of balanced 6% hydroxyethyl starch (HES) 130/0.4 did not result in reduced fluid requirement in severe burn victims.

● There is no advantage to using 6% HES 130/0.4 in severe burn injury.

## Abbreviations

ARDS: Acute respiratory distress syndrome; CI: Confidence interval; HES: Hydroxyethyl starch; ICU: Intensive care unit.

## Competing interests

TAN and RS have occasionally been participants of Fresenius Kabi (Stans, Switzerland) and B. Braun (Melsungen, Germany) nutrition and fluid therapy advisory board meetings and have received travel and accommodation support and also limited honoraria. MB has received travel and accommodation support and also limited honoraria (Fresenius Kabi and B. Braun). MF, MAP, and RAS declare that they have no competing interests.

## Authors’ contributions

RAS and MF collected the majority of the data and drafted parts of the manuscript. MAP performed statistical analysis. RS helped to analyze and interpret the data and drafted parts of the manuscript. MB and TAN led the project, collected parts of the data, performed additional statistical analysis, and drafted major parts of the manuscript. All authors read and approved the final manuscript.
